# Predictors of High Resource Consumption in Alcohol Intoxicated Patients in the Emergency Department

**DOI:** 10.3390/ijerph17114122

**Published:** 2020-06-09

**Authors:** Katharina Rönz, Trevor Hirschi, Sebastian Becker, Gert Krummrey, Aristomenis K. Exadaktylos, Thomas C. Sauter, Wolf E. Hautz, Martin Müller

**Affiliations:** 1 Department of Emergency Medicine, Inselspital, Bern University Hospital, University of Bern, 3010 Bern, Switzerland; Sebastian.Becker@insel.ch (S.B.); gert.krummrey@insel.ch (G.K.); aristomenis.exadaktylos@insel.ch (A.K.E.); thomas.sauter@insel.ch (T.C.S.); wolf.hautz@insel.ch (W.E.H.); martin.mueller2@insel.ch (M.M.); 2 Department of Emergency Medicine, Lindenhofspital, 3012 Bern, Switzerland; 3 Department of Anaesthesiology, Inselspital, Bern University Hospital, University of Bern, 3010 Bern, Switzerland; Trevor.hirschi@insel.ch; 4 Medical Skills Lab, Universitätsmedizin Berlin, 12203 Charité Berlin, Germany; 5 Institute of Health Economics and Clinical Epidemiology, University Hospital of Cologne, 50935 Cologne, Germany

**Keywords:** alcohol intoxication, alcoholism, emergency departments utilisation, emergency admissions

## Abstract

*Background:* previous studies have reported that the incidence of alcohol-related visits to emergency departments (ED) has increased, but little is known about how the necessary resources per visit have changed, or about the predictors and reasons for resource consumption. *Methods:* a retrospective analysis was performed of all consultations with a primary or secondary diagnosis of acute alcohol intoxication admitted to the ED of Bern University Hospital, Switzerland, between 1 June 2012, and 31 May 2017. Clinical characteristics and resource consumption were extracted and analysed over time. *Results:* in all, 196,045 ED consultations included 2586 acute alcohol intoxications, corresponding to 1.3% of the total. The incidences of acute alcohol intoxications have tended to increase over the last five years, and a growing number of visits have consumed high resources (consultations above the 75th percentile for total resource consumption). High resource consumption was associated with greater age and the male gender (*p* < 0.001). The main predictors of resource consumption were fractures (Odds ratio (OR): 3.9, 95% CI 2.8–5.3, *p* < 0.001), dislocations (OR 3.7, 95%: 1.5–9.1, *p* < 0.001), and traumatic brain injury (3.5, 2.5–5.1, *p* < 0.001). Consultations consuming high resources mostly required radiology resources (45%); consultations consuming low or normal resources mostly required physicians’ work (45%) or nurses’ work (27%). *Conclusions:* the number of alcohol intoxications consuming high resources has increased over the last five years. Acute alcohol intoxication associated with trauma is resource intensive, especially with regard to radiology resources. This underlines the need for further efforts to prevent alcohol-related traffic accidents, for examples.

## 1. Introduction

In recent decades, the burden of alcohol intoxication—either alone or in mixed intoxications, defined as mixed intoxication with alcohol and prescription drugs and/or illicit drugs—has continuously increased in Western countries, and remains a major and avoidable burden in emergency care [1,2,3,4].

Europe continues to have the highest levels of alcohol consumption in the world, and the highest share of all deaths attributable to alcohol consumption [5]. In 2016, the global average of estimated pure alcohol consumption per person aged 15 or older was 6.4 L. European (9.8 L), American (8.0 L), and Western Pacific countries (7.3 L) had higher consumption per person aged 15 or older than the global average, whereas African (6.3 L), South-East Asian (4.5 L), and Eastern Mediterranean countries (0.6 L) were below the global average [6]. In Switzerland, 9.4% of the population consumes alcohol every day (men: 12.5%, women 6.5%) and 50.9% once in a week (men 61.0%, women 41.2%) [7]. In 2016, the prevalence of chronic alcohol consumption was 4.3% of the population, defined as >40 g per day of pure alcohol for men and >20 g per day of pure alcohol in women [7]. The prevalence of daily, weekly, and chronic alcohol consumption was stable compared with 2011. In contrast, the prevalence of punctual alcohol consumption, defined as ≥4 standard drinks for women and ≥5 standard drinks for men (one standard drink: ca. 10–12 g of pure alcohol, e.g., 3 dL beer, 1 dL wine) has constantly increased in the group of 15 to 19-year-old adults between 2011 and 2014. In 2015, it decreased slightly but increased again in 2016 to 26.3% (2015: 25.0%). The group of 20 to 24-year-old adults showed the highest prevalence of punctual alcohol consumption with 38.2%. After that age, the prevalence decreases continuously [7].

The clinical presentation of patients with alcohol and mixed intoxications in the emergency department (ED) is heterogeneous, ranging from coma to aggressive states, and is often associated with trauma [3] Alcohol consumption is an independent risk factor for violence-related injuries and trauma at the ED [8,9,10]. In Switzerland, up to 15% of all injuries presenting at the ED have been estimated to be alcohol-related [11], but the true figure is thought to be higher, due to lack of routine testing [12]. Alcohol-related trauma is associated with increased trauma severity, mortality, and overall costs [13]. In addition, alcohol and especially mixed intoxication may lead to agitation and aggression, endangering the patient and others, and stressing the ED staff [14].

In 2010, alcohol-related costs in health care in Switzerland were 613 Mio Swiss Francs (~652 Million US Dollars) [15]. Health insurance is compulsory in Switzerland, which means that it provides cover for illness, maternity, and accidents, and offers the same range of services and benefits to all insured people. It is financed by policyholders’ contributions (premiums) and co-payments (deductible, retention fee, contribution to the costs of a hospital stay), and federal and cantonal funding (premium subsidies) [16].

A better understanding of the sociodemographic factors and ED resources needed by alcohol-intoxicated patients would help to ensure best medical practice, optimising planning of resources, reducing health insurance costs and encouraging authorities to implement preventive strategies and public campaigns.

In Japan, one study suggested that more ED resources are needed for alcohol-intoxicated cyclists, even after only a minor trauma [17]. However, there has been no detailed analysis of the need for different types of ED resources. In addition, the trend towards economic rationalisation in medicine demands better understanding of the predictors of high resource consumption, both overall and in particular diseases, in order to identify areas of resource scarcity and to estimate the impact of potential process optimisation.

Therefore, this study aims to (i) investigate the incidence of alcohol-intoxication in the ED population and to describe its characteristics, (ii) explore the detailed resource needs of ED patients with alcohol intoxication and their evolution in recent years, and (iii) identify predictors for high resource consumption in consultations by patients intoxicated with alcohol.

## 2. Materials and Methods

### 2.1. Study Design and Site

This is a retrospective analysis of all adult patients presenting with acute alcohol intoxication between 1 June 2012 and 31 May 2017 at the ED of Bern University Hospital (Inselspital). A total of more than 40,000 consultations each year have been documented at the ED in recent years [18].

### 2.2. Data Collection and Eligibility Criteria

There is no blood alcohol concentration cut-off to diagnose alcohol intoxication, as the individual response to alcohol intake varies widely between individuals and drinking habits [19]. According to the Diagnostic and Statistical Manual of Mental Disorders, version IV, acute alcohol intoxication is the combination of recent alcohol ingestion and a typical clinical presentation, such as slurred speech, lack of coordination, behavioural changes, or stupor [20]. Thus, in this retrospective study, we relied on the diagnosis made by the attending physician who treated a potentially eligible patient.

When a patient presents at our ED, the attending physician documents the diagnosis, history, medication, clinical findings, diagnostic measures, course of stay, and discharge procedure in a comprehensive electronic medical report. Furthermore, all health care professionals involved in the treatment—such as laboratory staff, nurses, radiology staff, physicians, and specialists—document their work in procedural codes for billing purposes. All health care staff are given regular training in the documentation of the correct procedural codes and these are rechecked for every patient by specially trained persons in our ED. The procedural codes are taken from the Tarmed Suisse catalogue—the tariff system in health care throughout Switzerland to label and assess medical services [21].

Eligible consultations were identified using a search algorithm based on key words—“intoxication”, “alcohol”, “ethanol”, etc., combined with the Boolean operator “OR” and with different semantic variations. This provided a highly sensitive algorithm to detect the patients based on the diagnostic or medical history fields in our computerised patient database (E-Care, ED 2.1.3.0, Turnhout, Belgium).

All adult patients (≥16 years) were eligible for study inclusion after a consultation led to the diagnosis of acute alcohol intoxication. We excluded consultations without a primary or secondary diagnosis of acute alcohol intoxication, duplicate records, incomplete data sets without documentation of the procedural codes or medical records, consultations of patients who were primarily seen by a psychiatrist (different billing system), and consultations of patients who did not give general consent for the use of their data in research. During the study period, there were no major changes in the procedural codes and databases used at our ED.

### 2.3. Data Extraction

The complete medical reports of all identified patients were extracted from the patient database. After duplicates had been removed, the medical history and patient diagnosis were screened in full text by hand, in order to establish the diagnosis of acute alcohol intoxication.

The following variables were coded by hand after analysing the medical reports in full text or were extracted from the computerised patient database:

- demographic data such as age, sex, and nationality;
- breath alcohol concentration, estimated blood alcohol concentration, or laboratory variables to estimate the blood alcohol concentration, i.e., sodium, potassium, urea, glucoses, and osmolality [22];
- signs of mixed intoxication;
- clinical presentation, i.e., any signs of aggression (verbal or physical), vital signs including the Glasgow Coma Scale (GCS), trauma grouped by fractures, traumatic brain injuries, cerebral bleeding, flesh or abrasion wounds, contusion, dislocation, and others (e.g., abdominal bleeding);
- treatment at the ED—intubation for respiratory compromise or police attendance needed;
- discharge procedure, such as emergency surgery, outpatient setting, or hospitalisation, as well as initial type of referral (walk-in, ambulance etc.).

For the outcome, selected procedural codes were grouped in the following categories by a committee of acute care nurses and ED physicians, in collaboration with the controller (SB) of our ED department:

- physicians’ work (including patient time and administrative time and costs);
- nurses’ work (including patient time and administrative time and costs);
- material expenses (e.g., injections, infusion, bandages, costs);
- laboratory resources (number, costs);
- radiology resources (ultrasound, computed tomography, X-rays, magnetic resonance imaging);
- total work or resources (sum of the above).

For all included patients, all selected procedural codes as well as sociodemographic and administrative data (i.e., time in the ED, and the need for hospitalisation and intermediate care unit (ICU) admission) were extracted from the administrative patient database (OpenText Suite for SAP^®^ Solutions, OpenText Corp., Waterloo, Canada).

In this study, the unit used for resource consumption is the Swiss medical currency “Tax points” (TP). In contrast to the total costs of a patient, TPs are more stable over time and directly reflect the “consumed” resources of a patient. At our hospital, one TP corresponds approximately to 0.86 Swiss Francs (about $0.87 US).

For resource comparison, patients were separated into two groups, with either high resource consumption—defined as being above the 75th percentile—or medium/low resource consumption—defined as being at the 75th percentile or below.

### 2.4. Ethical Considerations

The regional ethics committee of the Canton of Bern, Switzerland, classified the study as a quality assurance project and waived the need for informed patient consent and full ethical review according to Swiss law (Kantonale Ethikkomission (KEK): Req-2017-00454).

### 2.5. Statistical Analysis

The analysis was performed with Stata^®^ 13.1 (StataCorp, The College Station, TX, USA). Because normal distribution could not be ensured for most of the variables, all continuous variables are shown as medians with 25–75th interquartile ranges (IQR). Categorical variables are shown as per cent (absolute number).

Mann–Whitney U tests were performed to compare different interval variables for the two groups of resource consumption. The chi-square test was used to test for an association between categorical variables. Univariate associations were used to identify predictors of high resource consumption, with a stepwise forward multivariate logistic regression including all variables identified in univariate analysis with *p* < 0.1. A stepwise linear regression was used to model the total resource consumption based on clinical data. A *p*-value of less than <0.05 was considered significant.

## 3. Results

**Figure 1:**
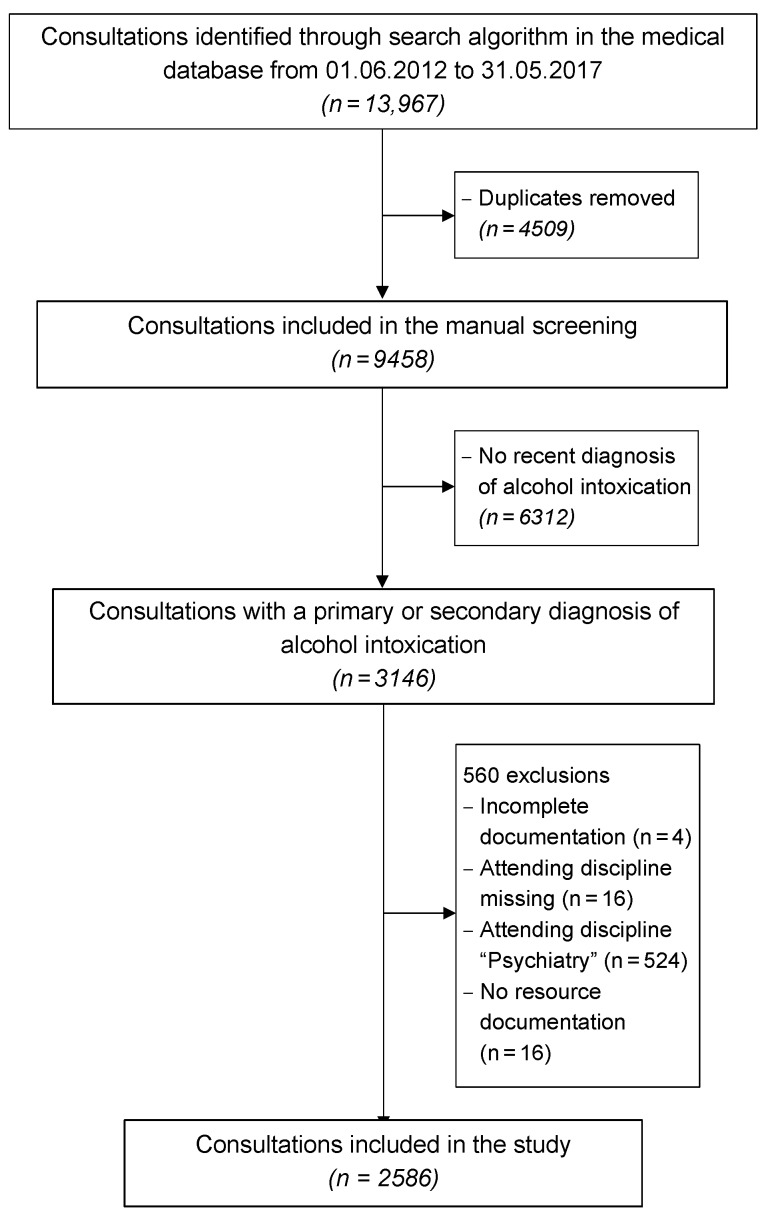
Flowchart of the study.

As shown in Figure 1, 13,967 out of 196,045 consultations in the medical database were identified through the search algorithm. Of these, 4509 consultations were duplicates and therefore excluded. The remaining 9458 consultations were manually screened. A total of 6312 consultations were excluded because there was no recent diagnosis of alcohol intoxication (e.g., past diagnosis only). Of the 3146 consultations with a primary or secondary diagnosis of alcohol intoxication, 560 consultations were excluded, either because the documentation was incomplete (*n* = 4), or the attending discipline was missing (*n* = 16), or the attending discipline was “Psychiatry” (*n* = 524) and/or resources were not documented (*n* = 16).

**Figure 2:**
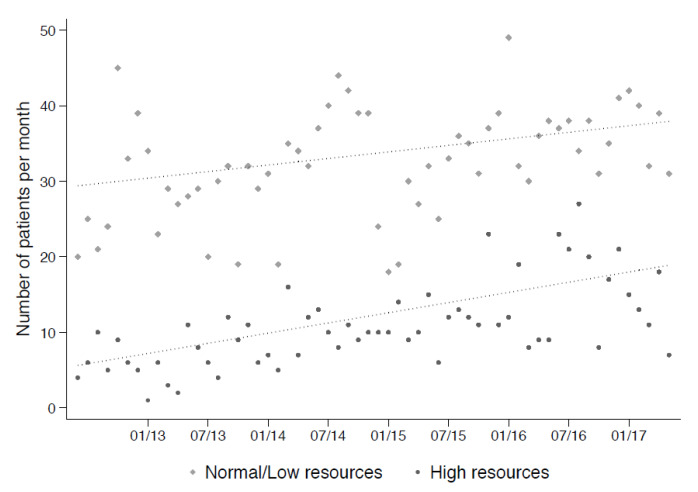
Number of alcohol intoxications per month over the course of the study, according to type of resource consumption.

Thus, 2586 consultations were included in the study. The median total resource consumption of these consultations was 1200 Tax Points (TP, medical currency), with an interquartile range ranging from 790–1901 TP. Moreover, 646 consultations had at least 1901 TP and were defined as high resource consumption consultations. There was a trend over the study period towards an increase in the total number of alcohol intoxications per month (median over the first six months of the study period in 2013 was 31 cases per months and the median was 55 cases per months in the last six months of the study period in 2017), with a greater increase in the number of consultations with high resource consumption (Figure 2).

### 3.1. Consultation Characteristics

Table 1 shows the characteristics of all included consultations grouped by high (*n* = 646) vs. low/normal (*n* = 1940) resource consumption.

The patients in the category of high resource consumption were significantly older (median 47, IQR 31–60 vs. median 35, IQR 24–50, *p* < 0.001), with a higher proportion of males (71.4% vs. 61.0%, *p* < 0.001). The intoxication was mixed in over one quarter (25.4%) of the high resource and 34.8% of the low/normal resource consultations (*p* < 0.001).

There were significant associations found between type of admission and type of resource consumption: the predominant admission type was via ambulance (70.2% in total, 74.8% of all the high resource consultations and 68.7% of all low/normal resource consultations, *p* = 0.003), followed by walk-in patients (17.7%) and admission through the police (5.9%). The air rescue admitted 4.8% of all high resource patients and 0.3% of all low/normal resource consultations (*p* < 0.001).

In the high resource group, a significantly (*p* < 0.001) greater number of consultations had a life threatening or urgent condition (20.1% high resource vs. 4.0% low/normal resource, or 43.3% vs. 35.0%).

A higher proportion of consultations with high rather than low/normal resource consumption were treated in the trauma room—a special high equipped room of the emergency department where instable and severe injured patients are treated (32.8% vs. 3.0%, *p* < 0.001) and hospitalised (43.5% vs. 28.8%, *p* < 0.001).

The clinical characteristics according to type of resource consumption are given in Supplementary Table S1.

### 3.2. Resource Consumption

**Figure 3:**
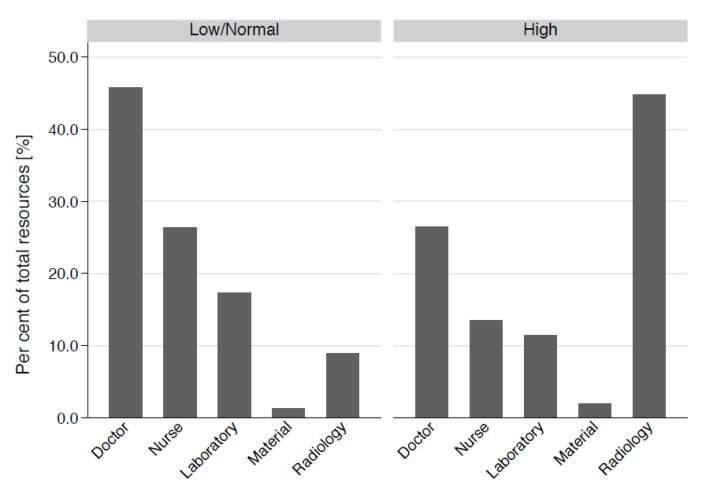
Comparison of the distribution of the total work according to the different resource categories according to type of resource consumption.

In the group with high resource consumption, the median total work was 2615 TP (IQR 2219–3490)—compared to 986 TP (IQR 679–1314) in the group of low/normal resource consumption (*n* = 1940), with an overall total work of 1200 TP (IQR 790–1901). The distribution of the total resources in shown in Table 2 and Figure 3. In all categories of resource consumption, i.e., physicians’ work (patient time, admin time, and report time), nurses’ work (patient time, and other nurses’ work), material expenses, laboratory (in TP and numbers), as well as radiology resources (total, ultrasound, X-ray, CT and MRI in TP and numbers), the consultations with high resource consumption exhibited significantly (*p* < 0.001) greater resource consumption than the consultations with normal/low resource consumption.

### 3.3. Predictors of Resource Consumption

The univariate associations between the clinical characteristics and high resource consumption are shown in Table 3.

Stepwise logistic regression revealed the following predictors of high resource consumption (Table 4A): Fractures had the highest odds ratio for high resource consumption (Odds ratio (OR) 3.9, 95% CI 2.8–5.3, *p* < 0.001), followed by dislocation (OR 3.7, 95% CI 1.5–9.1, *p* = 0.006), and traumatic brain injury (OR 3.5, 95% CI 2.4–5.1, *p* < 0.001). Other predictors were GCS ≤ 9, aggressive state, contusion, flesh and abrasion wounds and intubation. Suicidal intent was associated with a reduced odds ratio for high resource consumption (OR 0.6, 95% CI: 0.4–0.8, *p* = 0.003).

The identified parameters were confirmed in the analysis modelling total resource consumption using multivariate linear regression (Table 4B). The highest coefficient was found in patients with fractures (925 TP, 95% CI 761–1060) followed by traumatic brain injury (672 TP, 95% CI 518–826), and GCS ≤ 9 (653 TP, 95% CI 490–816)—all *p* < 0.001.

## 4. Discussion

Our analysis of patients with acute alcohol intoxications revealed a proportional incidence at our ED of 1.3% over the whole study period. In recent years, both consultations overall and (particularly) those consuming high resources have both tended to increase. High resource consumption was associated with higher age and male gender. Consultations with high resource consumption consumed a higher proportion of radiology resources than did consultations with low/normal resource consumption; the latter consultations mostly consumed physicians’ resources. The main predictors of high consumption of resources and of total resources were intoxications with associated trauma as well as factors leading to reduced clinical accessibility of the intoxicated patient.

### 4.1. Proportional Incidence and Characteristics

At first sight, the proportional incidence of 1.3% of acute alcohol intoxications found in our ED seems rather low in comparison to the numbers reported elsewhere [23]. In their tertiary referral hospital in the UK, the latter authors found that every fifth patient was admitted to the emergency department due to an alcohol-related problem. However, in contrast to our study, the authors included health problems related to chronic alcohol abuse, which were three times more common than acute alcohol intoxications. Another study from northeast England demonstrated a prevalence of alcohol-related ED admissions of 15% with a peak on weekends and in the early morning and a high rate of injuries [24]. In Belgium, 1.2% of all ED admissions were caused by alcohol intoxication, with a mean cost of 541 Euro per patient [3].

Between 2012 and 2017, we found an increase in ED visits related to alcohol intoxication, especially of visits that consumed high resources. In contrast, a study of the drinking behaviour of adolescents from 28 European and North American countries from 2002–2010 recorded a decline in weekly alcohol use in the United Kingdom and in Northern, Western, and Southern European countries [2]. According to official reports, alcohol consumption in Switzerland has remained stable in recent years, at a value of about 8.1 L of pure alcohol per inhabitant [25].

In our study, two thirds of patients with acute alcohol intoxication were male; the male gender showed an association with higher resource use. This is consistent with the findings for all WHO regions, where females are less often reported to be current drinkers than males. In addition, it was shown that women drink less than men. Young males are prone to be at risk of violence-related ED visits under alcohol intoxication and of trauma in general [26]. Previous studies showed a positive linear relation between alcohol and aggression in both men and women—up to a dose of 1.0 g/kg [3].

In 2016, 28.7% of all deaths attributable to alcohol consumption worldwide were due to injuries [1]. As alcohol abuse is associated with an increased number of comorbidities, it is not surprising to see that older patients in our study needed more resources.

We found that patients with mixed intoxication used fewer resources than patients with pure alcohol intoxication. One possible explanation for this may be that the additional drugs, e.g., benzodiazepines or opioids, reduce potential aggression because of their sedating effects.

### 4.2. Need of Resources

Human resources are mainly employed by consultations with low/normal resource consumption, and radiology resources by consultations with high resource consumption. This finding may be linked to the high incidence of trauma associated with alcohol intoxication. Several studies have found that alcohol consumption is a leading risk factor for mortality and morbidity—from both intentional and unintentional injury [8]. Traffic accidents in intoxicated patients are the most common unintentional injuries, followed by an accidental fall or trip.

### 4.3. Predictors and Risk Factors

In multivariate modelling, all factors that may make patient assessment difficult were associated with high use of resources, e.g., aggressive patients, reduced GCS < 9, intubated patients, or traumatic brain injury. All these factors warrant the use of technical investigations (radiology resources) to replace physicians’ judgment when treating these patients. Suicidal intent was associated with low/normal resource consumption (OR 0.6, 95% CI: 0.4–0.8, *p* = 0.003), as suicidal, intoxicated patients are just routinely monitored until they sober up and are transferred to psychiatry at our ED. In addition to this, all intoxications associated with trauma led to higher resource consumption when radiological imaging is performed; for example, a cranial CT scan in intoxicated patients with head trauma is recommended by guidelines [27].

The prediction model identified in our study needs further validation, but it could be a useful tool for calculating cost caused by alcohol-intoxicated patients.

In addition to direct costs investigated in our study, patients admitted for alcohol intoxication are at increased risk of social difficulties and mental health disorders [28]. It is therefore important to extend prevention programs for alcohol use to diminish the direct and indirect costs caused by alcohol-intoxicated patients. Public health programs and interventions are urgently required and may help to solve this problem [29].

Other preventive options may range from brief emergency room interventions to reduce re-admittance due to alcohol intoxications [30] to interventions at the national level by increasing taxes on alcoholic beverages [1], as a recent meta-analysis of the literature found that prices and taxes for alcoholic beverages are inversely related to the amount of alcohol consumption [4]. A disease modelling study suggested that violence-related ED visits might be reduced by 6000 a year in the UK by a 1% increase in the alcohol tax [29].

### 4.4. Limitations

The retrospective study design is a main limitation of the study and information bias cannot be excluded. Furthermore, diagnostic bias cannot be determined as we relied on the medical diagnosis of the attending physician for the diagnosis of acute alcohol intoxication. In addition, when blood alcohol level was incorporated into the regression model, it was not a predictor for resource utilization.

These biases are most likely equally distributed between all groups and are therefore not likely to influence the results of our study. A broad search algorithm with manual screening of the medical diagnoses was performed to further minimise selection bias. In addition, the outcome data are thought to be of high quality, as monthly training, lectures, and feedback are given to ensure appropriate and correct accounting in the ED. Those are particularly addressed towards new employees as part of the induction into the ED. Other departments (laboratory, radiology, ICU, and administrative data) also have validated procedures to ensure correct attribution of costs. Lastly, we present data over a 5-year period of time, which covered a great number of cases. However, our study included only data from a single university hospital in Bern, Switzerland. Thus, the external validity of our findings might be reduced.

## 5. Conclusions

In recent years, the incidence of ED consultations related to alcohol intoxication has increased. This applies particularly to visits consuming high resources and warrants interventions on all levels, from targeted ED interventions to large-scale efforts, such as tax adjustments. Intoxications with associated trauma are resource consuming (especially of radiological resources). This underlines the need for further preventive measures, e.g., to reduce alcohol-related traffic accidents.

## Figures and Tables

**Table 1:** Consultation characteristics of the group of patients (*n* = 2586).

Characteristic	Total (*n* = 2586)	High (*n* = 646)	Low/Normal (*n* = 1940)	*p*
Mixed intoxication, (*n* (%))	840 (32.5)	164 (25.4)	676 (34.8)	*<0.001*
Blood alcohol concentration *, (median (IQR))	1.6 (1.1–2.2)	1.6(1.2–2.2)	1.6 (1.1–2.2)	0.422
Sex, (*n* (%))				
Male	1645 (63.6)	461 (71.4)	1184 (61.0)	*<0.001*
Age, (median (IQR))	38 (26–52)	47 (31–60)	35(24–50)	*<0.001*
Type of admission, (*n* (%))				
Ambulance	1815 (70.2)	483 (74.8)	1332 (68.7)	*0.003*
General Practitioner	24 (0.9)	9 (1.4)	15 (0.8)	0.155
External Hospital	100 (3.9)	27 (4.2)	73(3.8)	0.634
Police	153 (5.9)	22 (3.4)	131 (6.8)	*0.002*
Air Rescue	37 (1.4)	31 (4.8)	6(0.3)	*<0.001*
Walk-In	457 (17.7)	74 (11.5)	383 (19.7)	*<0.001*
Triage, (*n* (%))				
Life-threatening	208 (8.0)	130 (20.1)	78 (4.0)	*<0.001*
Urgent conditions	959(37.1)	280 (43.3)	679 (35.0)	*<0.001*
Semi-urgent conditions	1293 (50.0)	201 (31.1)	1092 (56.3)	*<0.001*
Non-urgent conditions	74 (2.9)	11 (1.7)	63 (3.2)	*0.041*
Missing	52 (2.0)	24 (3.7)	28 (1.4)	*<0.001*
Discipline, (*n* (%))				
Internal medicine	1721 (66.6)	224 (34.7)	1497 (77.2)	*<0.001*
Surgery	853 (33.0)	422 (65.3)	431 (22.2)	*<0.001*
Fast-Track	12 (0.5)	0 (0.0)	12 (0.6)	*0.045*
Trauma room, (*n* (%))	270 (10.4)	212 (32.8)	58 (3.0)	*<0.001*
Discharge, (*n* (%))				
Outpatient treatment	1747 (67.6)	365 (56.5)	1382 (71.2)	*<0.001*
Hospital admission	839(32.4)	281 (43.5)	558 (28.8)	*<0.001*

Abbreviation: IQR: interquartile range; * Approximated blood alcohol concentration in g/kg available for 83.8% of the consultations; Significant *p*-values (<0.05) are highlighted in italic.

**Table 2:** Resource consumption of alcohol-intoxicated consultations (*n* = 2586).

Resource Group	Total (*n* = 2586)	High (*n* = 646)	Low/Normal (*n* = 1940)	*p*
Total work (TP)	1200 (790–1901)	2615 (2219–3490)	986 (679–1314)	<0.001
Physicians’ work				
Total physicians’ work (TP)	465 (329–633)	712 (539–929)	415 (309–535)	<0.001
Patient time (TP)	160 (115–231)	231 (169–329)	151 (107–195)	<0.001
Admin time (TP)	160 (89–231)	249 (160–355)	124 (89–195)	<0.001
Report time (TP)	39 (39–71)	71 (39–103)	39 (39–71)	<0.001
Nurses’ work				
Total nurses’ work (TP)	290 (164–374)	360 (275–433)	270 (124–348)	<0.001
Nurse patient time (TP)	255 (105–300)	285 (209–345)	235 (88–285)	<0.001
Nurse other effort (TP)	35 (0–93)	62 (35–98)	35 (0–93)	<0.001
Material expenses (TP)	9 (5–25)	35 (17–74)	8 (4–11)	<0.001
Laboratory resources				
Total laboratory effort (TP)	195 (88–330)	315 (176–441)	155 (76–293)	<0.001
Blood taken (yes), *n* (%)	2214 (85.6)	628 (97.2)	1586 (81.8)	<0.001
Radiology resources				
Total effort radiology (TP)	0 (0–746)	1147 (851–1820)	0 (0–164)	<0.001
Ultrasound, *n* (%)	269 (10.4)	210 (32.5)	59 (3.0)	<0.001
X-ray, *n* (%)	1126 (43.5)	628 (97.2)	498 (25.7)	<0.001
CT, *n* (%)	875 (33.8)	566 (87.6)	309 (15.9)	<0.001
MRI, *n* (%)	50 (1.9)	47 (7.3)	3 (0.2)	<0.001

Abbreviation: CT: Computer tomography; MRI: Magnet resonance imaging; TP: Tax points, medical currency (1 TP worth approximately 0.86 Swiss Francs). The distributions are shown as median (IQR) if not indicated otherwise.

**Table 3:** Univariate association between clinical characteristics and high resource consumption.

High Resource Consumption	Odds Ratio	95% Conf. Interval	*p*
Circumstances			
Age, (per year)	1.03	1.03, 1.04	<0.001
Sex, (male)	1.59	1.31, 1.93	<0.001
Mixed intoxication, (yes)	0.64	0.52, 0.78	<0.001
Suicidal intent, (yes)	0.49	0.35, 0.71	<0.001
Aggressive, (yes)	1.03	0.71, 1.49	0.882
GCS ≤ 9, (yes)	2.52	1.84, 3.46	<0.001
Accompanying injuries			
Fracture, (yes)	6.54	4.97, 8.6	<0.001
Traumatic brain injury, (yes)	6.92	5.05, 9.48	<0.001
Cerebral bleeding, (yes)	6.50	3.73, 11.33	<0.001
Dislocation, (yes)	5.03	2.36, 10.7	<0.001
Contusion, (yes)	3.98	2.88, 5.49	<0.001
Flesh wound, (yes)	3.79	3, 4.78	<0.001
Abrasion wound, (yes)	4.14	3.33, 5.13	<0.001
Blood alcohol concentration*, (per g/Kg)	1.00	0.89, 1.13	0.961
Procedure			
Police attendance, (yes)	0.90	0.72, 1.12	0.354
Emergency surgery, (yes)	3.30	2.33, 4.65	<0.001
Intubation needed, (yes)	5.45	2.93, 10.14	<0.001

Note: * Available for 83.8% of the consultations; GCS: Glasgow coma scale.

**Table 4:** (**A**) Predictors of high resource consumption through multivariate logistic regression and (**B**) predictors of total resource consumption though multivariate linear regression.

(A) High Resource Consumption	Odds Ratio	95% Conf. Interval	*p*
Age	1.03	1.03, 1.04	<0.001
GCS ≤ 9	2.83	1.89, 4.23	<0.001
Suicidal intent	0.56	0.38, 0.82	0.003
Aggressive	1.41	0.95, 2.11	0.090
Fracture	3.88	2.83, 5.32	<0.001
Traumatic brain injury	3.52	2.44, 5.06	<0.001
Dislocation	3.65	1.46, 9.13	0.006
Contusion	2.55	1.75, 3.70	<0.001
Flesh wound	1.78	1.23, 2.59	0.002
Abrasion wound	2.15	1.52, 3.04	<0.001
Intubation	2.04	0.90, 4.61	0.088
**(B) Total resources**	**Coefficient**	**95% Conf. Interval**	***p***
Age	12	10, 15	<0.001
Mixed intoxication	113	32, 193	0.006
GCS ≤ 9	653	490, 816	<0.001
Fracture	925	791, 1060	<0.001
Traumatic brain injury	672	518, 826	<0.001
Cerebral bleeding	249	−18, 515	0.067
Dislocation	329	−25, 683	0.068
Contusion	460	304, 617	<0.001
Flesh wound	142	−13, 297	0.073
Abrasion wound	338	193, 483	<0.001
Intubation	590	274, 906	<0.001

Abbreviation: GCS: Glasgow coma scale.

## Supplementary Materials

The following are available online at https://www.mdpi.com/1660-4601/17/11/4122/s1, Table S1. Clinical characteristics according to type of resource consumption.
