# Supplementary material for: Predictors of High Resource Consumption in Alcohol Intoxicated Patients in the Emergency Department
Source: Int J Environ Res Public Health. 2020 Jun 9;17(11):4122. doi: 10.3390/ijerph17114122 (PMC7312041; doi:10.3390/ijerph17114122)
Supplement: Supplementary file 1 [file ijerph-17-04122-s001.pdf]

Table S1. Clinical characteristics according to type of resource consumption.

Characteristic	Total (*n* = 2586)	High (*n* = 646)	Low/Normal (*n* = 1940)	*p*
Circumstances, *n* (%)				
Suicidal intent	256 (9.9)	38 (5.9)	218 (11.2)	<0.001
Aggressive	157 (6.1)	40 (6.2)	117 (6.0)	0.882
GCS ≤ 9	171 (6.6)	75 (11.6)	96 (4.9)	<0.001
Accompanying injuries, *n* (%)				
Fracture	253 (9.8)	160 (24.8)	93 (4.8)	<0.001
Traumatic brain injury	190 (7.3)	125 (19.3)	65 (3.4)	<0.001
Cerebral bleeding	58 (2.2)	39 (6.0)	19 (1.0)	<0.001
Dislocation	29 (1.1)	18 (2.8)	11 (0.6)	<0.001
Contusion	162 (6.3)	88 (13.6)	74 (3.8)	<0.001
Flesh wound	355 (13.7)	178 (27.6)	177 (9.1)	<0.001
Abrasion wound	433 (16.7)	219 (33.9)	214 (11.0)	<0.001
Other trauma	41 (1.6)	7 (1.1)	34 (1.8)	0.238
Procedure, *n* (%)				
Police attendance	558 (21.6)	131 (20.3)	427 (22.0)	0.354
Emergency surgery	139 (5.4)	70 (10.8)	69 (3.6)	<0.001
Intubation needed	44 (1.7)	28 (4.3)	16 (0.8)	<0.001

Abbreviation: GCS: Glasgow coma scale.
